# Opinions towards Companion Animals and Their Welfare: A Survey of Croatian Veterinary Students

**DOI:** 10.3390/ani10020199

**Published:** 2020-01-24

**Authors:** Tomislav Mikuš, Mario Ostović, Ivana Sabolek, Kristina Matković, Željko Pavičić, Ornella Mikuš, Željka Mesić

**Affiliations:** 1 Department of Hygiene, Technology and Food Safety, Faculty of Veterinary Medicine, University of Zagreb, Heinzelova 55, 10000 Zagreb, Croatia; tmikus@vef.hr; 2 Department of Animal Hygiene, Behaviour and Welfare, Faculty of Veterinary Medicine, University of Zagreb, Heinzelova 55, 10000 Zagreb, Croatia; isabolek@vef.hr (I.S.); kmatkov@vef.hr (K.M.); zpavicic@vef.hr (Ž.P.); 3 Department of Agricultural Economics and Rural Development, Faculty of Agriculture, University of Zagreb, Svetošimunska cesta 25, 10000 Zagreb, Croatia; omikus@agr.hr; 4 Department of Marketing in Agriculture, Faculty of Agriculture, University of Zagreb, Svetošimunska cesta 25, 10000 Zagreb, Croatia; zmesic@agr.hr

**Keywords:** animal welfare, survey, companion animals, cats, dogs, veterinary students

## Abstract

**Simple Summary:**

Although veterinarians are expected to have the main role in ensuring the welfare of all animals, it has been established that their perception of animal welfare may vary regarding animal species observed. To the best of our knowledge, there is no literature report on a comprehensive study focusing solely on veterinary attitudes and opinions towards companion animal welfare. The present study included students of veterinary medicine in Croatia and revealed them to have strongly positive opinions, with minor oscillations, towards companion animals and their welfare throughout the study years. Study results can contribute to the knowledge of veterinary perception of animal welfare and their opinions and attitudes towards welfare of different animal species, including companion animals and welfare challenges nowadays encountered in these species.

**Abstract:**

This survey was the first one investigating opinions of veterinary students in Croatia towards companion animals and their welfare, with special reference to dogs and cats as the most popular companion animals in the European Union. The study included students of all six years of the integrated undergraduate and graduate veterinary medicine study programme in Croatia. First-year students were surveyed twice, before and after having attended the course on animal welfare. Student opinions were assessed on the basis of their mean responses to five-point Likert scale questions and frequency of responses to Yes/No/I do not know questions and ratio scale questions. Study results revealed students to have strongly positive opinions towards companion animals and their welfare. The majority of student statements did not differ significantly between the first and sixth study years or before and after having attended the animal welfare course in the first study year, mostly yielding a straight, non-fluctuating line. Students were not sure whether welfare of companion dogs and cats was compromised. Study results pointed to reliable and reasonable opinions of veterinary medicine students in Croatia towards companion animals and their welfare, as well as to the welfare issues these species may be facing nowadays.

## 1. Introduction

Companion animals (CA) have been playing an increasingly important role in modern human life. By definition, CA include all species that humans choose to share their lives and homes with [1]. CA are considered to have a primarily social role in the household or community, thus being distinguished from working or production animals. The term CA is based on human perception of the role and value of a particular animal rather than on the intrinsic quality of the animal itself [2].

The number of CA in the Western world has abruptly increased in the past few decades, with more than 50% of households owning one or more animals [3]. In the European Union (EU), the most common CA species is cat with a population of approximately 75.3 million (M), followed by dog (65.5 M), ornamental birds (35.6 M), small mammals (19.4 M), aquaria (10.6 M) and reptiles (6.3 M) [4].

The ever-greater number and variety of CA certainly pose new challenges and require additional efforts from veterinary profession. According to the latest Eurobarometer survey on the attitudes of Europeans towards animal welfare [5], the majority of EU citizens (74%) including Croatian ones (79%) believe that CA welfare should be better protected.

Veterinarians are expected to have a crucial role in ensuring and promoting high animal welfare standards [6,7]. However, the perception of animal welfare among veterinarians may vary depending on the level of education and professional practice [8]. As animal welfare is a relatively young field of science [9], the education following this science is still being actively developed. Veterinary curricula are known to differ all over the world [10,11], provoking differences in the knowledge and skills, as well as attitudes and opinions of students, veterinarians-to-be, towards animal welfare.

Therefore, studies assessing attitudes and opinions of veterinary students and veterinarians towards animal welfare have been gaining importance, as confirmed by numerous papers published in recent years. Our previous study [12] showed generally concerned attitudes of veterinary students in Croatia towards farm animal welfare. However, upon detailed analysis of their attitudes, it appeared that they did not think rationally about the issue of farm animal welfare, as they might perceive these animals just as food production animals, implying their practical value; this in particular held true for final-year students [13]. Lower attitudes towards animals and their welfare in veterinary students at higher study years were also recorded in other studies [6,14,15,16]. In addition, previous studies showed veterinary students to express more positive attitudes and opinions towards CA welfare than towards farm animal welfare [17,18,19,20]. We wondered whether it also held true for Croatian veterinary students. The more so, searching the literature, we found no comprehensive study on the attitudes and opinions of veterinary students focused solely on different CA welfare issues.

Therefore, we embarked upon this study to investigate the opinions of veterinary students in Croatia towards CA and their welfare, and to see whether and how their opinions were modified over study years.

## 2. Materials and Methods

### 2.1. Participants/Sample

The survey was conducted at the Faculty of Veterinary Medicine, University of Zagreb, which is the only veterinary faculty in Croatia. Students of all six years of the integrated undergraduate and graduate veterinary medicine study programme were surveyed. First-year students were surveyed twice, before and after having attended the compulsory 40-h Environment, Animal Behaviour, and Welfare course. This yielded total response rates of 93% (n = 513 students) and 91% (n = 505 students), respectively. Students of all six study years had the same curriculum on animal welfare. The survey was voluntary, anonymous and approved by the institutional Board for Quality Management (for details, see Ostović et al. [12]).

### 2.2. Questionnaire

The written questionnaire was composed of two parts (as presented in the Supplementary Materials). The first part included student demographics, i.e., age, sex, secondary school completed, early environment, owning or keeping companion animals, and preferred/chosen study track. The second part referred to 32 statements used to examine their opinions towards CA and their welfare, with special reference to dogs and cats as the predominant CA in EU. These statements were in the forms of five-point Likert scale questions (1 = fully disagree, 2 = disagree, 3 = neutral/unsure, 4 = agree, and 5 = fully agree), Yes/No/I do not know questions, and ratio scale questions. The questionnaire was pre-tested [12], with reliability (α) of 0.683.

### 2.3. Statistical Analysis

Statistical data analysis was performed by use of SPSS v. 21.0 software. The frequencies of student responses were calculated by use of univariate analysis. Assessment of student opinions was based on the frequency of their responses to the Yes/No/I do not know questions and ratio scale questions, and their mean responses to the Likert scale questions. First-year student responses recorded after having attended the course on animal welfare were taken into consideration on calculating total mean scores (the mean values across all study years). Wilcoxon signed-rank test was used on testing differences between first-year student responses to Likert scale questions before and after having attended the course on animal welfare, whereas differences in responses among all study years were tested by Kruskal–Wallis test and Mann–Whitney *U*-test. The value of *p* < 0.05 was considered significant in all tests.

## 3. Results

Study results are presented descriptively, graphically and in tables. Data analysis revealed that student responses to the majority of Likert scale questions varied among study years; yet, summarizing the results obtained pointed to a particular pattern in student responses. Therefore, in order to present student responses to Likert scale questions across study years as clearly as possible, we decided to report only first- and sixth-year student responses, and first-year student responses before and after having attended the course on animal welfare. The total mean score still included responses of students of all six study years but without responses of first-year students before attending the course on animal welfare. The responses to the Likert scale questions given by students of all study years are presented in the Supplementary Materials.

**Figure 1:**
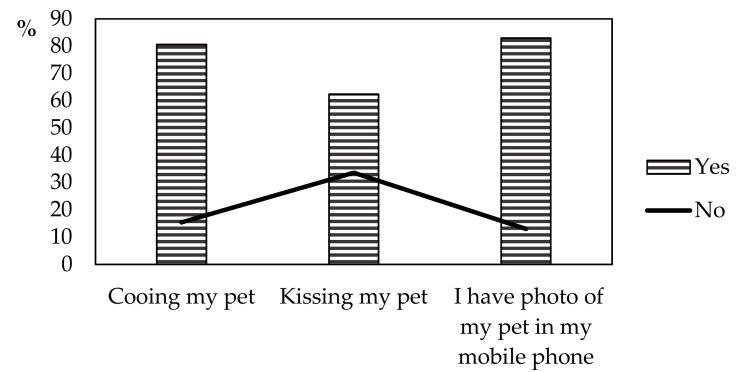
Student relationship (n = 505) with their own pet.

Demographic student profile according to study years was shown in our previous study [12]. Briefly, 75.2% were females; 59.5% were aged 18–21 years; 82.3% had completed high school; 74.9% had urban background; 95.3% owned or kept CA; and more than half of the subjects (54%) preferred/chose CA study track. More than 60% of students treated their CA in a parental manner (Figure 1), while 62.6% of student families allocated up to 50 € per month for CA feed.

Students agreed that owning CA had a favourable impact on human health and taught children to be responsible; therefore, according to their opinion, education on CA should be included in kindergarten schedule. Students also considered that pet owners sometimes acted against CA welfare and compromised their welfare due to the lack of knowledge while meaning well. They were not sure whether each family should keep CA and whether their owners were well informed on CA and its needs before taking it. Students had neutral opinions towards the owners keeping more than one CA species and their ability to take due care of all their CA, as well as on the issue of achieving the same level of emotional bonding with different CA species. In addition, students believed that CA and farm animals should be equally treated.

Upon having attended the course on animal welfare, first-year students were significantly less likely (W = 652, Z = -2.371, *p* = 0.02) to indicate that children should be educated on CA in kindergartens, but significantly more likely (W = 3551, Z = -2.309, *p* = 0.02) to state that the owners of more than one CA species could take due care of all their CA species. Last-year students also expressed significantly less positive opinions (U = 3518, Z = -2.458, *p* = 0.02) concerning the issue of children education on CA, but higher opinions (U = 3357, Z = -2.578, *p* = 0.01) on the pet owners occasionally acting against CA welfare deliberately, as compared with first-year students before the course on animal welfare. Moreover, last-year students showed a significantly lower rate of agreement on the issue of pet owners acquiring adequate information on CA and its housing, feeding, and care, as compared with first-year students before (U = 2895, Z = -3.776, *p* = 0.00) and after the course on animal welfare (U = 2866, Z = -3.355, *p* = 0.00). Considering other statements listed in Table 1, there were no significant differences in opinions between first-year students before and/or after the course on animal welfare and sixth-year students.

**Figure 2:**
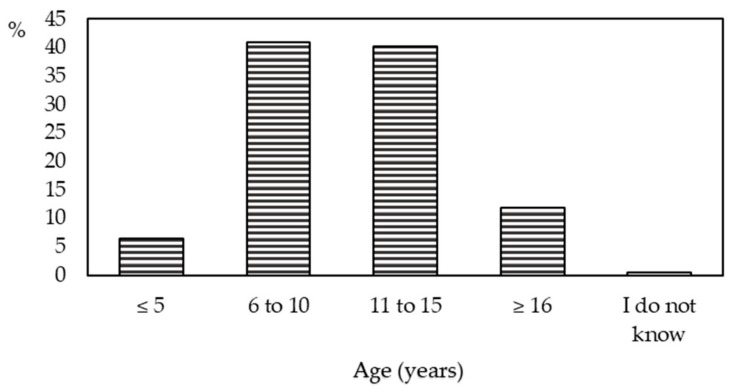
Age at which students (n = 505) believe children should be given a pet to take care of.

Students believed that CA should not be given to children younger than 5 years but at an older age, between 6 and 15 years (Figure 2).

Half of the study students (50.5%) considered that pet owners kept pedigree dogs or cats for profit, whereas 81% of them were ready to take a dog or cat from animal shelter, in which they would also like to work (62.4%); 64.2% of students were against the animal shelter kill policy; 64.6% of students considered CA exhibitions stressful for animals; and 58.6% of students were concerned about welfare of dogs and cats in commercial breeding establishments.

Students of all study years believed that both dogs and cats were animals with high cognitive abilities; yet, students were not sure whether their welfare was compromised when kept as CA. Last-year students were significantly less likely to believe that dogs were capable to think (U = 3785, Z = -2.040, *p* = 0.04) and have emotions (U = 3908, Z = -2.033, *p* = 0.04) but were significantly more likely (U = 3611, Z = -2.099, *p* = 0.04) to believe that cats could have emotions, as compared with first-year students before the course on animal welfare. There were no significant differences between first-year student opinions towards the level of cognitive abilities in either dogs or cats before and after the course on animal welfare. Also, considering their welfare compromise, no significant differences in first-year student opinions before and after the course on animal welfare, or between first- and sixth-year students were recorded (Table 2).

Students considered routine castration of dogs and cats justifiable but dog ear cropping and tail docking, cat declawing and dog tethering as cruel practices. There were no significant differences in student responses to these statements in first-year students before and after the course on animal welfare, or between first-year and sixth-year students, with a note that the latter evaluated castration as a significantly more justified practice when compared with first-year students either before (U = 3082, Z = -3.350, *p* = 0.00) or after the course on animal welfare (U = 2477, Z = -4.540, *p* = 0.00). Eating dogs and cats was unacceptable to Croatian veterinary students, yet significantly less unacceptable (U = 3458, Z = -2.409, *p* = 0.02) to last-year students as compared with first-year students before the course on animal welfare (Table 3).

## 4. Discussion

Teaching veterinary students is not just an educational exercise but has a substantial role in the formation and learning of these professionals how to cope with the emotionally difficult aspects of veterinary work, as well as in the development of their attitudes and opinions towards animal welfare as the most important task of each veterinary practitioner [13,21,22]. To our knowledge, the present study is the first one addressing the opinions of Croatian veterinary students towards CA and their welfare.

Study results indicated that the greatest proportion of students preferred/chose the CA study track, which could be explained by their mostly urban background and previous experience with these animals, as almost all study subjects owned or kept CA. The majority of students reported cooing and kissing their pets, having their photos in mobile phones, and spending up to 50 € monthly for pet feed. Serpell [23] reports on identical findings, suggesting that interactions with animals have strong influence on the development of values in veterinary students. Izmirli et al. [24] also found keeping CA to be strongly associated with moral values in veterinary students, their decision to study veterinary medicine and their satisfaction with veterinary curriculum. Similar results on owning CA by veterinary students have been reported by Magnani et al. [19] as well. Another reason for veterinary students aspiring at having their career focused on CA could be that the study is predominated by female students who are mainly familiar with CA and strive to work with species other than farm animals [20]. The more so, considering the livestock reduction in Croatia [25], veterinary students can be expected to choose the CA segment of veterinary medicine, which has a stable foundation and is developing at a fast rate. Accordingly, more than 60% of our students recognized shelter medicine as their potential career. It is no surprise because it is a rapidly growing discipline of veterinary medicine [26,27]. The readiness of students to work in animal shelter points to their high level of empathy for homeless animals (more than 80% of students were willing to take a shelter animal) and the challenges encountered in this activity, while suggesting that students have been properly prepared for labour market because shelter work is very demanding, both emotionally and professionally. A shelter veterinarian is expected to be very skilful in many fields of veterinary medicine, such as emergency care, internal medicine, surgery, infectious diseases, behavioural health and epidemiology [27,28].

Owning a pet is known to teach children to be responsible and affects their attachment, resulting in children’s positive attitudes towards animals later in life [29,30,31]. As the majority of negative attitudes towards animals initially develop in early periods of life, educational programmes should foster the knowledge and positive attitudes towards animals already in kindergarten children [32]. Our students agree with this opinion but considering that pets and care for them should be delegated to children only at the age of 6 or even later, which is in line with other recent studies. Almeida et al. [33] suggest that children aged 8–10 years tend to misinterpret the meaning of particular animal behaviours. Muldoon et al. [34] also found younger children in the 7–13 age group to be focused mostly on facial expressions of CA while being capable of recognizing only if their animal is hungry, whereas older children emphasized emotional distress associated with experiences such as loneliness or homesickness.

Our students support the statement that keeping CA has many benefits for mental and physical health of their owners, as confirmed by numerous reports (e.g., [35,36,37,38]). They neither agree nor disagree whether each family should have CA, which is consistent with their neutral opinion on the issue whether CA owners acquire adequate information on the respective CA and its needs and whether the owners of more than one CA species can take care of all their CA properly. Indeed, it may eventually lead to the CA failing to meet the owner’s expectations, impaired pet welfare and irresponsible pet ownership, breaking the owner-animal relationship, and at the worst animal relinquishment. Previous studies have pointed to the need for targeted education of the potential pet owners to ensure their expectations be aligned with the reality of pet ownership [39] and to reduce relinquishments [40]. For example, Plitman et al. [41] found that owners of brachycephalic cats were less inclined to get due information prior to buying the cat and that, once owning it, they did not consider their cats healthy (in particular due to their skin and eye condition), as compared with the owners of other pedigree cats. These owners also recommended their breed to other people less frequently, probably because of the poor health experiences and/or high maintenance requirements.

Our students believe that pet owners sometimes act against animal welfare out of ignorance, or occasionally even deliberately compromise their welfare; however, the line between these two categories may frequently be hardly perceivable. Pet feeding is a typical example referring to both these issues. Many owners feed their pets with inappropriate food and/or excessive amounts of food, thus predisposing obesity as the most common nutritional disorder in dogs and cats [42,43,44], major welfare issue and One Health problem [45,46]. In their report, Kipperman and German [47] argue that it is veterinarian duty and responsibility to address CA obesity.

The reason to have a pedigree CA can also be profit for their owners [48], and half of our veterinary students agree with this statement. In addition, they believe that current pedigree dog and cat breeding practices raise many welfare concerns, which has been extensively discussed lately all over the world [49,50,51]. Pedigree animals may appear in breed shows, which can be stressful for animals [52], as justifiably stated by the students as well. It has been reported that noise, novelty, transport, training, immobilization or restricted housing conditions can act as stressors on animals, eliciting responses in behavioural, cardiovascular, endocrine, renal, gastrointestinal and haematological parameters [53].

Students expressed neutral opinions on whether the same level of emotional attachment could be established with different CA species. The study by Mueller [54] showed the human to animal relationship to vary depending on animal species, and the animal species owned by the person to influence his/her attitudes concerning emotional attachment with different animal species. As reported by Borgi and Cirulli [55], the ability of CA to bond with humans is considered as meeting the human need for attention and emotional attachment, thus sharing similar psychological and adaptive functions as human-human friendship. Sevillano and Fiske [56] investigated how humans perceived different animal species regarding warmth and competence. Dogs were rated as the warmest and most competent species. In the study by Phillips et al. [57], dog was also perceived as a species of high sentience, being ranked just after human infant and chimpanzee. Moreover, the study by Smolkovic et al. [58] revealed dog owners to be more attached to their pets as compared to cat owners. The reason for such a specific human-dog bond might be found in the social cognitive abilities that humans and dogs may share, in particular, visual cues in communication [59,60,61].

In the study by Magnani et al. [19], veterinary students ranked the level of dog and cat welfare highest among all animal species investigated; yet, along with ruminants, these species raised most animal welfare concerns in students. The authors explained it by the previously reported veterinary student perception of the high cognitive abilities in dogs and cats [17]. Our students also firmly believe that dogs and cats are animals with high cognitive abilities but are indecisive when asked about their welfare compromise in general. This finding may suggest the arising welfare challenges in companion dogs and cats, faced by veterinary students in Croatia.

Levine et al. [17] found veterinary students to consider various procedures including surgical procedures performed on animals more humane for farm mammals than for dogs and cats. Comparison of the results obtained in the current study with those recorded in our previous study on farm animals [12] reveals similar opinions in Croatian veterinary students; in addition, our students consider the procedures performed on poultry to be more humane as compared with dogs and cats. However, our students consider routine castration of dogs and cats a justifiable surgical procedure, which is consistent with the results of the study conducted by Hedge et al. [62].

Our veterinary students consider ear cropping and tail docking in dogs cruel procedures. Cropping and docking are prohibited in many countries as these practices are found unnecessary, painful and cruel [63,64], although illegal cases still exist [65]. As reported by Sinmez et al. [63], it will take time to modify mentality of a subgroup of veterinarians about the issue, to become the true and caring professionals and defenders of animal welfare and veterinary societies. Declawing in cats is another controversial animal welfare issue [66,67], also considered cruel procedure by our students. Moreover, students also consider dog tethering a cruel practice. In terms of welfare, penning provides no improvement either [68]. According to the Animal Protection Act in the Republic of Croatia [69], dog ear cropping and tail docking, and cat declawing are prohibited procedures, except for medical reasons and in hunting dogs, in line with cynology standards. Besides this, continuous dog tethering and penning without ensuring free movement are prohibited. Furthermore, with the above-mentioned Act, no kill policy in animal shelters has been adopted, except for justifiable cases, as also supported by the majority of our veterinary students.

In Croatia, killing dogs and cats for food and other products is prohibited [69], and their consumption in some parts of the world is not acceptable to our students. Differences in consuming various animal species in particular parts of the world can be attributed to cultural variations that are known to affect human perception [57,70], including veterinary students [24] of animals and their welfare.

Significant differences in opinions towards CA and their welfare found in this study were recorded more frequently between first-year and last-year students than in first-year students before and after having attended the course on animal welfare, mostly with lower response values in final-year students. However, for example, final-year students ranked the statement that pet owners sometimes acted against CA welfare significantly higher, suggesting that students gathered experience and knowledge about CA throughout their study in spite of the potential empathy decline occurring in veterinary students at higher study years, known as emotional hardening [6,14,15,16]. Nevertheless, there were no significant differences in opinions on the majority of statements including opinions towards general welfare compromise in dogs and cats in first-year students before and after the course on animal welfare or between first-year and last-year students. This finding shows that veterinary student opinions towards CA and their welfare were constant throughout the study.

Students agree with the public opinion [5] that CA and farm animals, in general, deserve equal treatment concerning animal welfare. A similar finding has been reported by Serpell [23]. However, comparing current results with those obtained in a previous study [12], it appears that now they have more positive opinions towards CA.

## 5. Conclusions

The results of this survey suggest that veterinary students in Croatia have strongly positive opinions towards CA and their welfare. They actually maintained such opinions from the very beginning to the end of their study, with some minor oscillations, indicating stability of their opinions irrespective of education and related experiences, along with their great interest to work in this field of veterinary medicine. It is supported by the finding that students were not sure whether welfare of companion dogs and cats was compromised, suggesting that they identified and thought about welfare issues that nowadays can also involve these species. The results obtained can contribute to the knowledge of veterinary opinions and attitudes towards animal welfare and their perception of welfare issues in different animal species, including companion animals. The investigation was conducted as a descriptive, cross-sectional study. To obtain more accurate results, a longitudinal study including the same students from the beginning to the end of their study should be performed. In addition, further studies should include gauging behaviours and values (i.e., affective abilities) such as empathy to measure their attitudes and investigate the association between variables and their effect on student attitudes and behaviours.

## Figures and Tables

**Table 1:** Student opinions towards general statements on companion animals.

Statement	Study Year			Total Score ^TS^ (n = 505)
	First ^A^ (n = 143)	First ^B^ (n = 135)	Sixth (n = 60)	
	Mean * (SD)			
Keeping CA is beneficial for human health	3.92 (1.26)	4.04 (1.12)	4.25 (1.10)	4.32 (1.02)
Each family should have a CA	3.35 (1.47)	3.28 (1.33)	2.95 (1.49)	3.42 (1.38)
Keeping CA teaches children to be responsible	4.39 (0.86)	4.32 (0.94)	4.03 (1.16)	4.35 (0.93)
Children should be educated on CA in kindergartens	4.65 ^a^ (0.61)	4.44 ^b^ (0.89)	4.22 ^b^ (1.12)	4.41 (0.91)
Before taking a CA, owners are thoroughly informed on CA and its needs	3.18 ^a^ (1.40)	3.14 ^a^ (1.46)	2.40 ^b^ (1.38)	3.01 (1.46)
Owners sometimes act against the CA welfare	3.92 ^a^ (0.94)	4.04 (1.04)	4.25 ^b^ (0.99)	4.12 (0.97)
Owners sometimes compromise CA welfare, meaning well but due to the lack of knowledge	4.12 (0.76)	4.11 (1.02)	4.15 (0.92)	4.12 (0.94)
Owners of more than one CA species can take due care of all their CA	2.53 ^a^ (1.18)	2.81 ^b^ (1.32)	2.45 (1.35)	2.82 (1.26)
The same level of emotional bonding can be achieved with all CA species	2.87 (1.37)	2.81 (1.28)	2.90 (1.39)	2.95 (1.34)
CA deserve better treatment than farm animals	2.25 (1.27)	2.28 (1.40)	2.13 (1.26)	2.36 (1.36)

CA—companion animal; A—answered before taking the course on animal welfare; B—answered after the course; * 1—fully disagree; 5—fully agree; TS—calculated as the mean of all study year values including second, third, fourth and fifth years but excluding first year before attending the course on animal welfare; a,b—values in the same row labelled by different letters differed significantly (*p* < 0.05).

**Table 2:** Student opinions towards the level of cognitive abilities in dogs and cats and their welfare compromise.

Statement		Study Year			Total Score ^TS^ (n = 505)
		First ^A^ (n = 143)	First ^B^ (n = 135)	Sixth (n = 60)	
		Mean * (SD)			
Thought process	Dogs	4.83 ^a^ (0.46)	4.83 (0.45)	4.67 ^b^ (0.66)	4.76 (0.56)
	Cats	4.42 (0.84)	4.53 (0.81)	4.62 (0.59)	4.54 (0.77)
Emotions	Dogs	4.93 ^a^ (0.28)	4.85 (0.43)	4.80 ^b^ (0.51)	4.80 (0.54)
	Cats	4.48 ^a^ (0.70)	4.53 (0.77)	4.65 ^b^ (0.76)	4.53 (0.82)
Welfare compromise	Dogs	2.99 (1.12)	3.24 (1.23)	3.20 (1.23)	3.09 (1.23)
	Cats	2.90 (1.09)	3.11 (1.11)	3.00 (1.15)	2.90 (1.16)

A—answered before taking the course on animal welfare; B—answered after the course; * 1—fully disagree; 5—fully agree; TS—calculated as the mean of all study year values including second, third, fourth and fifth years but excluding first year before attending the course on animal welfare; a,b—values in the same row labelled by different letters differed significantly (*p* < 0.05).

**Table 3:** Student opinions towards justifiability/cruelty of particular practices considering dog and cat welfare.

Statement	Study Year			Total Score ^TS^ (n = 505)
	First ^A^ (n = 143)	First ^B^ (n = 135)	Sixth (n = 60)	
	Mean * (SD)			
Routine castration of dogs and cats is justifiable	3.90 ^a^ (1.00)	3.69 ^a^ (1.05)	4.38 ^b^ (0.83)	4.01 (0.99)
Dog ear cropping and tail docking are cruel	4.06 (1.04)	4.13 (0.99)	3.90 (1.31)	4.07 (1.12)
Cat declawing is cruel	4.47 (0.81)	4.38 (0.85)	4.17 (1.26)	4.33 (0.99)
Dog tethering is cruel	4.51 (1.00)	4.50 (1.01)	4.38 (1.20)	4.42 (1.06)
Dog and cat consumption is cruel	4.18^a^ (1.20)	3.90 (1.34)	3.77 ^b^ (1.29)	3.93 (1.36)

A—answered before taking the course on animal welfare; B—answered after the course; * 1—fully disagree; 5—fully agree; TS—calculated as the mean of all study year values including second, third, fourth and fifth years but excluding first year before attending the course on animal welfare; a,b—values in the same row labelled by different letters differed significantly (*p* < 0.05).

## Supplementary Materials

The following are available online at https://www.mdpi.com/2076-2615/10/2/199/s1.
