# Supplementary material for: Opinions towards Companion Animals and Their Welfare: A Survey of Croatian Veterinary Students
Source: Animals (Basel). 2020 Jan 24;10(2):199. doi: 10.3390/ani10020199 (PMC7070996; doi:10.3390/ani10020199)
Supplement: Supplementary file 1 [file animals-10-00199-s001.pdf]

Opinions towards Companion Animals and Their Welfare: A Survey of Croatian Veterinary Students

Supplementary Material 1:

1. Study year: 1 2 3 4 5 6
2. Preffered/chosen study track: a) Companion animals b) Farm animals and horses c) Veterinary public health d) I do not know
3. Gender: a) Male b) Female
4. Age (years): ___________
5. Early environment: a) Rural b) Urban
6. Secondary school: a) High school b) Veterinary school c) Other ___________
7. Have you owned or kept companion animals? a) Yes b) No
8. If your answer to question 7 is Yes, please mark the following statements accordingly:
   I coo my pet a) Yes b) No
   I kiss my pet a) Yes b) No
   I have photo of my pet in my mobile phone a) Yes b) No
9. If your answer to question 7 is Yes, please mark the amount of money allocated by your family for pet feed per month: a) < 50 € b) 50–100 € c) > 100 €
10. According to your opinion, at what age should be children given a pet to take care of? __________
11. Please indicate your level of agreement/disagreement with the following statements (5 – fully agree, 4 – agree, 3 – neutral/unsure, 2 – disagree, 1 – fully disagree):

Keeping companion animal (CA) is beneficial for human health	5	4	3	2	1
Each family should have a CA	5	4	3	2	1
Keeping CA teaches children to be responsible	5	4	3	2	1
Children should be educated on CA in kindergartens	5	4	3	2	1
Before taking a CA, owners are thoroughly informed on CA and its needs	5	4	3	2	1
Owners sometimes act against the CA welfare	5	4	3	2	1
Owners sometimes compromise CA welfare, meaning well but due to the lack of knowledge	5	4	3	2	1
Owners of more than one CA species can take due care of all their CA	5	4	3	2	1
The same level of emotional bonding can be achieved with all CA species	5	4	3	2	1
CA deserve better treatment than farm animals	5	4	3	2	1

12. Please indicate your level of agreement/disagreement with the statement that the following species are capable to think (5 – fully agree, 4 – agree, 3 – neutral/unsure, 2 – disagree, 1 – fully disagree):

Dogs	5	4	3	2	1
Cats	5	4	3	2	1

13. Please indicate your level of agreement/disagreement with the statement that the following species are capable of having emotions (5 – fully agree, 4 – agree, 3 – neutral/unsure, 2 – disagree, 1 – fully disagree):

Dogs	5	4	3	2	1
Cats	5	4	3	2	1

14. Please indicate your level of agreement/disagreement with the statement that welfare of the following species is compromised (5 – fully agree, 4 – agree, 3 – neutral/unsure, 2 – disagree, 1 – fully disagree):

Dogs	5	4	3	2	1
Cats	5	4	3	2	1

15. Would you consider working in an animal shelter? a) Yes b) No c) I do not know
16. Would you adopt a dog or cat from animal shelter? a) Yes b) No c) I do not know
17. Do you find pet owners of pedigree dogs or cats to have them for profit? a) Yes b) No c) I do not know
18. Do you find pet exhibitions stressful for animals? a) Yes b) No c) I do not know
19. Do you agree with an animal shelter kill policy? a) Yes b) No c) I do not know
20. Do you find dog and cat welfare compromised in commercial breeding establishments? a) Yes b) No c) I do not know

21. Please indicate your level of agreement/disagreement with the following statements (5 – fully agree, 4 – agree, 3 – neutral/unsure, 2 – disagree, 1 – fully disagree):

Routine castration of dogs and cats is justifiable	5	4	3	2	1
Dog ear cropping and tail docking are cruel	5	4	3	2	1
Cat declawing is cruel	5	4	3	2	1
Dog tethering is cruel	5	4	3	2	1
Dog and cat consumption is cruel	5	4	3	2	1

*The questionnaire also included questions related to welfare of farm animals and game animals. Study results on those species have been published elsewhere.

Table S1. Student opinions towards general statements on companion animals.

	Study Year							Total score[TS] (n = 505)
	First[A] (n = 143)	First[B] (n = 135)	Second (n = 108)	Third (n = 74)	Fourth (n = 73)	Fifth (n = 55)	Sixth (n = 60)	
	Mean* (SD)							
Keeping CA is beneficial for human health	3.92 (1.26)	4.04 (1.12)	4.66 (0.79)	4.47 (0.82)	4.36 (0.99)	4.18 (1.17)	4.25 (1.10)	4.32 (1.02)
Each family should have a CA	3.35 (1.47)	3.28 (1.33)	3.64 (1.32)	3.78 (1.41)	3.47 (1.32)	3.26 (1.43)	2.95 (1.49)	3.42 (1.38)
Keeping CA teaches children to be responsible	4.39 (0.86)	4.32 (0.94)	4.69 (0.54)	4.38 (0.89)	4.21 (1.07)	4.27 (0.93)	4.03 (1.16)	4.35 (0.93)
Children should be educated on CA in kindergartens	4.65 (0.61)	4.44 (0.89)	4.69 (0.68)	4.43 (0.88)	4.19 (0.97)	4.24 (0.98)	4.22 (1.12)	4.41 (0.91)
Before taking a CA, owners are thoroughly informed on CA and its needs	3.18 (1.40)	3.14 (1.46)	3.08 (1.49)	2.92 (1.42)	3.34 (1.46)	2.87 (1.36)	2.40 (1.38)	3.01 (1.46)
Owners sometimes act against the CA welfare	3.92 (0.94)	4.04 (1.04)	4.21 (0.88)	4.20 (0.92)	3.96 (0.99)	4.11 (0.96)	4.25 (0.99)	4.12 (0.97)
Owners sometimes compromise CA welfare, meaning well but due to the lack of knowledge	4.12 (0.76)	4.11 (1.02)	4.33 (0.72)	4.16 (0.91)	3.90 (0.97)	3.87 (1.02)	4.15 (0.92)	4.12 (0.94)
Owners of more than one CA species can take due care of all their CA	2.53 (1.18)	2.81 (1.32)	2.75 (1.31)	2.84 (1.22)	3.15 (1.02)	2.96 (1.23)	2.45 (1.35)	2.82 (1.26)
The same level of emotional bonding can be achieved with all CA species	2.87 (1.37)	2.81 (1.28)	2.95 (1.40)	3.20 (1.40)	3.01 (1.31)	2.87 (1.25)	2.90 (1.39)	2.95 (1.34)
CA deserve better treatment than farm animals	2.25 (1.27)	2.28 (1.40)	2.02 (1.27)	2.60 (1.39)	2.67 (1.32)	2.76 (1.35)	2.13 (1.26)	2.36 (1.36)

CA – companion animal; A – answered before taking the course on animal welfare; B – answered after the course; *1 – fully disagree; 5 – fully agree; TS – calculated as the mean of all study year values excluding first year before attending the course on animal welfare.

Table S2. Student opinions towards the level of cognitive abilities in dogs and cats and their welfare compromise.

		Study Year							Total Score[TS] (n = 505)
		First[A] (n = 143)	First[B] (n = 135)	Second (n = 108)	Third (n = 74)	Fourth (n = 73)	Fifth (n = 55)	Sixth (n = 60)	
		Mean* (SD)							
Thought process	Dogs	4.83 (0.46)	4.83 (0.45)	4.87 (0.36)	4.77 (0.61)	4.63 (0.66)	4.64 (0.70)	4.67 (0.66)	4.76 (0.56)
	Cats	4.42 (0.84)	4.53 (0.81)	4.66 (0.70)	4.66 (0.67)	4.25 (0.95)	4.49 (0.77)	4.62 (0.59)	4.54 (0.77)
Emotions	Dogs	4.93 (0.28)	4.85 (0.43)	4.94 (0.23)	4.81 (0.57)	4.59 (0.81)	4.69 (0.64)	4.80 (0.51)	4.80 (0.54)
	Cats	4.48 (0.70)	4.53 (0.77)	4.65 (0.73)	4.62 (0.79)	4.18 (1.11)	4.55 (0.69)	4.65 (0.76)	4.53 (0.82)
Welfare compromise	Dogs	2.99 (1.12)	3.24 (1.23)	2.86 (1.19)	3.04 (1.29)	3.12 (1.28)	3.09 (1.13)	3.20 (1.23)	3.09 (1.23)
	Cats	2.90 (1.09)	3.11 (1.11)	2.70 (1.16)	2.80 (1.27)	2.70 (1.08)	3.04 (1.12)	3.00 (1.15)	2.90 (1.16)

A – answered before taking the course on animal welfare; B – answered after the course; *1 – fully disagree; 5 - fully agree; TS – calculated as the mean of all study year values excluding first year before attending the course on animal welfare.

Table S3. Student opinions towards justifiability/cruelty of particular practices considering dog and cat welfare.

	Study Year							Total Score[TS] (n = 505)
	First[A] (n = 143)	First[B] (n = 135)	Second (n = 108)	Third (n = 74)	Fourth (n = 73)	Fifth (n = 55)	Sixth (n = 60)	
	Mean* (SD)							
Routine castration of dogs and cats is justifiable	3.90 (1.00)	3.69 (1.05)	4.12 (1.06)	4.10 (0.89)	4.01 (0.98)	4.09 (0.82)	4.38 (0.83)	4.01 (0.99)
Dog ear cropping and tail docking are cruel	4.06 (1.04)	4.13 (0.99)	4.02 (1.24)	4.15 (1.04)	4.12 (1.04)	3.98 (1.18)	3.90 (1.31)	4.07 (1.12)
Cat declawing is cruel	4.47 (0.81)	4.38 (0.85)	4.49 (0.86)	4.46 (0.97)	4.18 (0.98)	4.07 (1.14)	4.17 (1.26)	4.33 (0.99)
Dog tethering is cruel	4.51 (1.00)	4.50 (1.01)	4.53 (0.90)	4.15 (1.28)	4.45 (0.97)	4.38 (1.11)	4.38 (1.20)	4.42 (1.06)

Dog and cat consumption is cruel	4.18 (1.20)	3.90 (1.34)	4.08 (1.35)	4.07 (1.37)	3.77 (1.56)	3.93 (1.17)	3.77 (1.29)	3.93 (1.36)

A – answered before taking the course on animal welfare; B – answered after the course; *1 – fully disagree; 5 –fully agree; TS – calculated as the mean of all study year values excluding first year before attending the course on animal welfare.
